# Ankylosis of the cervical spine increases the incidence of blunt cerebrovascular injury (BCVI) in CTA screening after blunt trauma

**DOI:** 10.1007/s10140-022-02022-8

**Published:** 2022-03-16

**Authors:** Riku M. Vierunen, Ville V. Haapamäki, Mika P. Koivikko, Frank V. Bensch

**Affiliations:** Department of Radiology, Helsinki University and Helsinki University Hospital, Töölö Hospital, Topeliuksenkatu 5, FIN–00029 Helsinki, Finland

**Keywords:** Ankylosing spondylitis, Blunt cerebrovascular injury, CT angiography, Cervical spine, Spinal fracture

## Abstract

**Purpose:**

To examine the incidence, location, and grade of blunt cerebrovascular injury (BCVI), as well as associated strokes in patients with ankylosis of the cervical spine, imaged with CT angiography (CTA) after blunt trauma. The related etiologies of ankylosis had an additional focus.

**Materials and methods:**

Altogether of 5867 CTAs of the craniocervical arteries imaged after blunt trauma between October 2011 and March 2020 were manually reviewed for a threshold value of ankylosis of at least three consecutive cervical vertebrae. BCVI was the primary outcome and associated stroke as the secondary outcome. Variables were craniofacial and cervical spine fractures, etiology and levels of ankylosis, traumatic brain injury, spinal hematoma, spinal cord injury, and spinal cord impingement, for which correlations with BCVI were examined.

**Results:**

Of the 153 patients with ankylosis and blunt trauma of the cervical spine, 29 had a total of 36 BCVIs, of whom two had anterior and 4 posterior circulation strokes. Most of the BCVIs (*n* = 32) were in the vertebral arteries. Injuries were graded according to the Biffl scale: 17 grade II, 4 grade III, 14 grade IV, and 1 grade V. A ground-level fall was the most common trauma mechanism. Cervical spine fracture was the only statistically significant predictor for BCVI (OR 7.44). Degenerative spondylosis was the most prevalent etiology for ankylosis.

**Conclusion:**

Ankylosis of the cervical spine increases the incidence of BCVI up to sevenfold compared to general blunt trauma populations, affecting especially the vertebral arteries.

## Introduction

High-energy deceleration forces from blunt trauma resulting in hyperflexion, hyperextension, or rotation of the neck can cause cervical arteries to stretch over or shear against adjacent structures, in addition to the direct impact caused by fractured bones. The resulting intimal damage can affect both carotid and vertebral arteries and is defined as blunt cerebrovascular injury (BCVI) [1]. BCVI has an incidence of 1% to 2.7% in blunt trauma [2–4] and up to 9.2% in hospitalized patients with severe trauma and traumatic brain injury (TBI) [5]. Untreated, BCVI can cause cerebral or cerebellar infarction via thromboembolism or vessel occlusion, which can be prevented by timely instituted anticoagulation [5–7]. While digital subtraction angiography is still considered the gold standard for the diagnosis of BCVI, CTA is the de facto standard of imaging, thanks to its speed, reliability, and cost-effectiveness [5, 7–12].

Spinal ankylosis carries a risk for unstable fractures, even from low-energy trauma, usually localized in the lower cervical spine [13]. HLA B27-associated ankylosing spondylitis (AS) usually develops under the age of 30 with a ratio of 2:1 men to women and 0.1% to 1.4% prevalence among European populations [14]. In the USA, prevalence among genders is approximately equal, with more severe radiological findings in men [15]. Chronic inflammatory processes cause ankylosis affecting predominantly sacroiliac joints and the cervicothoracic spine [14]. Other axial ankylosis-causing seronegative spondyloarthropathies are psoriatic arthritis, reactive arthritis, enteropathic-related spondyloarthritis, and undifferentiated spondyloarthritis [16].

Diffuse idiopathic skeletal hyperostosis (DISH) is defined as ankylosis of at least four adjacent vertebrae in the thoracic spine, usually presenting as anterior “flowing” syndesmophytes that tend to evade the pulsating aorta to the contralateral side [17–19]. Prevalence increases with age, reaching 35% in US men over 70, but is less frequent among Asian populations, or in general among women [20, 21]. While the precise etiology of the disease is unknown, its incidence correlates with obesity, type 2 diabetes, metabolic syndrome, and elevated insulin levels [22–24].

We hypothesize that ankylosis of the cervical spine leads to increased incidence of BCVI with less trauma energy compared to general blunt trauma populations. To our knowledge, no previous publications are focusing on the incidence of BCVI in the trauma of the ankylosed cervical spine. The purpose of this retrospective study was to examine the incidence, location, and Biffl grading of BCVI in the post-traumatic ankylosed cervical spine, with an additional focus on the related incidence of acute cerebral infarctions [25].

## Materials and methods

The University Hospital’s institutional review board approved this retrospective study.

### Patients and inclusion criteria

Töölö Hospital, a part of Helsinki University Hospital, is the only level-1 trauma center for a catchment area of 1.67 million people, where emergency whole-body CTs for blunt trauma are routinely performed. Patients with serious trauma such as cervical spine fractures and injuries are frequently transferred to our institution from general hospitals both outside of and within the same health care district for further evaluation and treatment. All patients over the age of 15, both primary and referrals with any blunt trauma and ankylosis of at least three consecutive cervical spine vertebrae were included. To our knowledge, studies in the literature have not established a cut-off value for significant ankylosis that predisposes patients to fractures. Patients under 16 years or without ankylosis of at least three consecutive cervical vertebrae, as well as patients with penetrating trauma were excluded. The Impax Picture Archiving and Communications System (Impax 6, Agfa Healthcare NV, Mortsel, Belgium) allowed manual retrieval and reassessment of all CTAs between October 2011 and March 2020.

### Imaging

The trauma imaging protocol involves an initial CTA of the craniocervical arteries from the aortic arch to skull base followed by a split-bolus whole-body CT from clavicles to ischium in both the arterial and the venous phase and includes a nonenhanced head CT. We use contrast media with a concentration of 350 mg/ml (Omnipaque 350, GE Healthcare, Milwaukee, WI, USA) in the split-bolus technique. The first bolus consists of 80 ml of contrast media with a flow rate of 5 ml/s, followed by a second bolus of 50 ml with a flow rate of 4 ml/s injected with a 40 s delay. If diagnosed with a BCVI, a follow-up CTA is routinely performed after two weeks of anticoagulation treatment. CTA of craniocervical arteries and CT and MRI scans for cerebral ischemia or disorders of the spinal cord are performed upon request for topical ischemic insult. All CT images were obtained using a 64-slice CT scanner (Discovery CT 750 HD, GE Healthcare, Milwaukee, WI), while MRIs were performed on a 1.5 T closed-bore MRI scanner (Signa LX 1.5 T, GE Healthcare, Milwaukee, WI). For CTAs, coronal and sagittal reformatted series with 2 mm slice thickness, and, for head CTs, axial, coronal, and sagittal reformatted series with 3 mm slice thickness are made in addition to the basic volumetric dataset of 0.625 mm slice thickness for all patients. For CTAs of craniocervical arteries, maximal intensity projection (MIP) images are reconstructed using a vascular kernel, as well as bone window reformats of the cervical spine and skull using a bone kernel. The MRI protocol for BCVI consists of an unenhanced time of flight magnetic resonance angiography (TOF MRA), as well as a T1-weighted, fat-saturated three-dimensional turbo spin-echo (3D TSE SPACE) sequence of the neck. The MRI protocol for the trauma of the cervical spine includes T- and T2-weighted TSE sequences in both sagittal and transverse planes as well as STIR in the sagittal and a fat-saturated T2-weighted sequence of the craniocervical junction in the coronal plane.

### Imaging analysis

During the evening and night, images are interpreted by an on-call resident, while an attending radiologist who is specialized or a fellow in trauma radiology double-read images by day. A board-certified radiologist with 5 months of experience as a fellow in trauma radiology (RV, Reader 1) reviewed all CTAs for ankylosis. Of the included patients, levels of fused segments, fractured cervical vertebrae, and intervertebral discs were recorded. A second board-certified trauma radiologist with 15 years of experience (VH, Reader 2) was blinded to the original reports and reassessed all included CTAs for the etiology of ankylosis. A third board-certified trauma radiologist with 10 years of experience (FB, Reader 3), along with Readers 1 and 2, reviewed all patients’ CTAs for BCVIs independently and blinded to one another, after which disagreements were settled by consensus. Documentation of BCVI comprised the affected artery, level of injury, and Biffl grading, as presented in Table 1 [25].

**Table 1:** Grading of BCVI according to Biffl et al. [25]

Injury grade	Description of BCVI
I	Luminal irregularity or dissection with < 25% luminal narrowing
II	Dissection or intramural hematoma with ≥ 25% luminal narrowing, intraluminal thrombus, or raised intimal flap
III	Pseudoaneurysm
IV	Total occlusion
V	Transection with free extravasation

*BCVI*, blunt cerebrovascular injury

### Variables

**Fig. 1:**
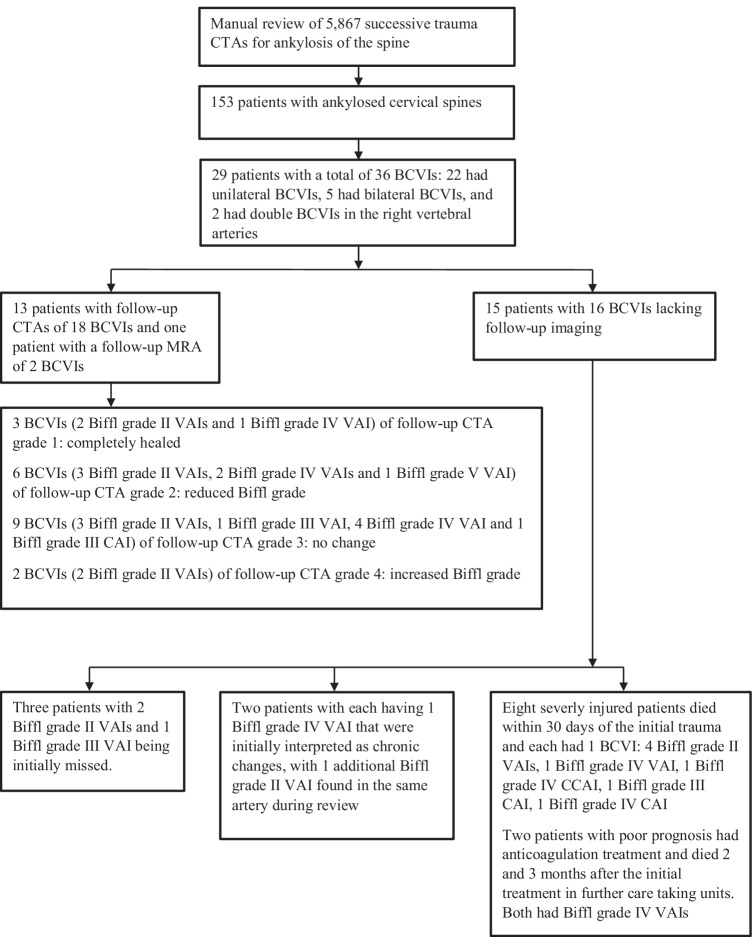
Flowchart of manually reviewed 5867 CTAs of craniocervical arteries after blunt trauma revealing 153 patients with ankylosis of at least three consecutive cervical vertebrae. The workflow shows 14 patients on the left who had follow-up CTAs and an MRA classified by grading shown in Table 2 and 15 patients on the right who did not have follow-up examinations. Those 15 who lacked follow-up imaging were divided into three categories: initially missed BCVIs, chronic deemed changes, and severely injured or those with poor prognosis. BCVI, blunt cerebrovascular injury; CTA, CT angiography; MRA, magnetic resonance angiography

The primary and secondary variables and outcomes were BCVI and associated acute stroke, respectively. The main predictive variables were fractures of the cervical spine, facial bones, and skull. Additional variables were age, gender, etiology and levels of ankylosis, trauma mechanism, and TBI. Spinal hematoma, spinal cord injury, and spinal cord impingement were recorded from MRIs, which were imaged upon request. TBI was defined as subdural, subarachnoideal, epidural, or intraparenchymal hemorrhage visible in head CTs or contusions visible only in MRIs. Furthermore, we recorded the development of BCVIs in follow-up CTAs and the 30-day mortality rate. Findings from follow-up CTAs regarding the progression of primarily detected BCVIs were evaluated (Table 2 and flow chart, Fig. 1). Original reports by radiologists and treating clinicians for the documentation of BCVI, ankylosis, management of anticoagulation therapy, and trauma mechanisms were reviewed. The etiology of ankylosis was defined as AS or seronegative spondyloarthropathy, DISH, degenerative spondylosis (DS), or surgical fusion [26].

**Table 2:** Grading of follow-up CTAs in patients with BCVI

Follow-up CTA grade	Description of follow-up CTA
1	Completely healed
2	Reduced Biffl grade
3	No change
4	Increased Biffl grade

*BCVI*, blunt cerebrovascular injury; *CTA*, CT angiography

### Statistical analysis

Associations between variables were analyzed as follows: cervical spine fracture versus BCVI (*χ*^2^ test), skull fracture versus BCVI (Fisher’s test), gender versus BCVI (*χ*^2^ test), and age versus BCVI (Mann–Whitney *U* test). Only those with one type of predictive fracture variable were subjected to the calculation for association with BCVI. The logistic regression model analyzed predictive and other variables (except age) to explain the relative risk for BCVI. The number of ankylosed spinal segments and the number of fractured vertebrae and transversely fractured intervertebral discs were evaluated for correlation using Spearman’s *ρ*. Interobserver agreement was calculated using Cohen’s *κ* between Reader 1 and Reader 2 for analysis of etiology in ankylosis and for BCVI findings between readers’ results and consensus reading. If a patient was diagnosed with the correct number of BCVIs, the reading was considered valid. Strength of agreement was defined as follows: values < 0, no agreement; 0.00–0.20, slight; 0.21–0.40, fair; 0.41–0.60, substantial; 0.81–1.00, almost perfect [27]. *P*-values < 0.05 were regarded as statistically significant. All statistical analyses were performed using SPSS v. 25 (IBM Corp., Armonk, NY).

## Results

### Primary and secondary outcomes

**Fig. 2:**
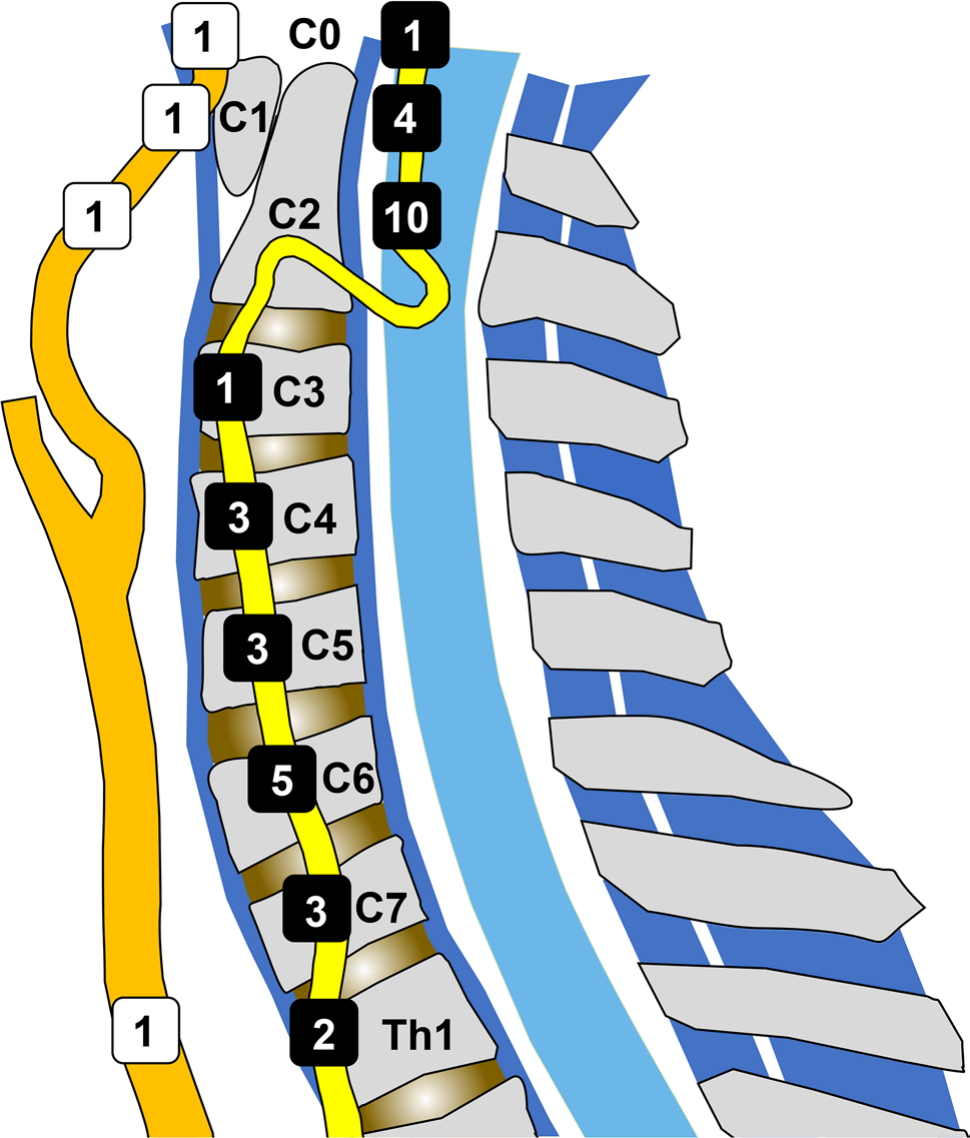
Distribution of 36 BCVIs in 29 patients in terms of cervical vertebral level. BCVIs in vertebral arteries are shown by white numbers within black squares and those in carotid arteries by black numbers within white squares. Common carotid and internal carotid artery are shown in the orange and vertebral artery in yellow. BCVI, blunt cerebrovascular injury

**Fig. 3:**
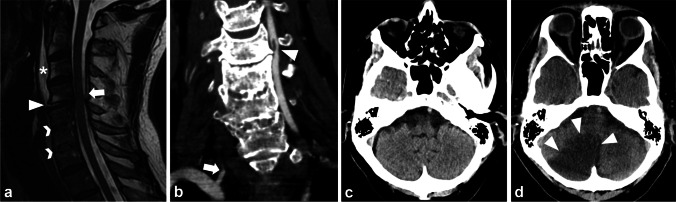
A 67-year-old patient with ankylosis from DS between the fifth and seventh cervical vertebrae after two anterior intervertebral disc implants between the fifth and sixth and the sixth and seventh cervical vertebrae suffered an impact from being struck by heavy construction scaffolding, resulting in an unstable fracture through the intervertebral disc between the fourth and fifth cervical vertebrae (arrowhead). T2w MRI in the sagittal plane reveals posterior epidural hematoma (arrow) and prevertebral hemorrhage (asterisk) (**a**). This CTA coronal image with 2 mm slice thickness shows a grade II BCVI with an intraluminal thrombus located in the left VA (arrowhead), while the right VA is occluded owing to a grade IV BCVI (arrow) (**b**). An unenhanced head CT taken in the acute phase showed no signs of acute ischemia (transverse reformat with 3 mm slice thickness (**c**). Two days after the initial injury, a cerebellar infarction is demarcated as a clearly hypodense area in transverse reformat with 3 mm slice thickness (**d**). BCVI, blunt cerebrovascular injury; CTA, CT angiography

**Fig. 4:**
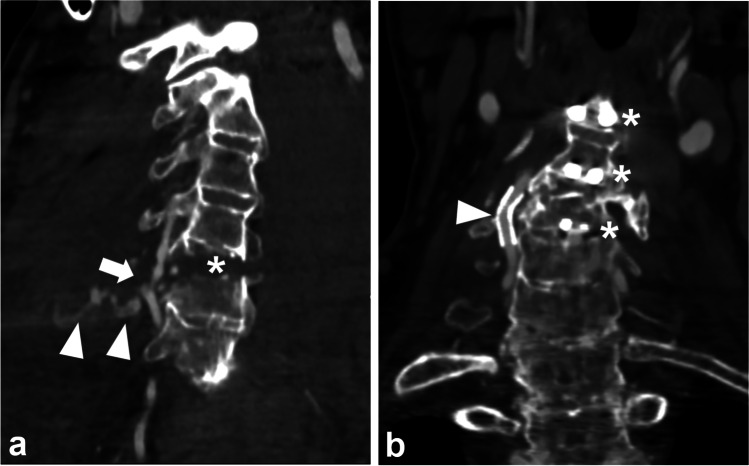
After falling from a bicycle, a patient aged 57 was found by his neighbor, developed swelling around the neck, and had difficulties in breathing caused by active extravasation (arrowheads) from a grade V BCVI in the right vertebral artery (arrow) shown in CTA of 2 mm thickness coronal slices. The unstable cervical spine fracture (asterisk) disrupted the intervertebral disc between the fourth and fifth cervical vertebrae in the ankylosed spine caused by ankylosing spondylitis (**a**). The BCVI was treated with an endovascular stent. On follow-up CTA, the stented vertebral artery (arrowhead) remained open without any ischemic lesions. Anterior fixation screws (asterisks) are shown in the third, fourth, and fifth vertebrae (**b**). BCVI, blunt cerebrovascular injury; CTA, CT angiography

Retrospective reassessment of 5867 CTAs after blunt trauma, imaged between October 2011 and March 2020 at our institution, revealed 153 patients (111 men, median age 75 years, interquartile range 67.5–82) with uniform ankylosis of at least three successive vertebrae, of whom 29 (19%) had 36 BCVIs. Of the 36 BCVIs (Fig. 2), 32 (89%) were in the vertebral arteries, three in internal carotid arteries, and one in the common carotid artery. Two patients had two vertebral artery injuries (VAIs) in the right vertebral artery, four had bilateral VAIs, and one had a right VAI with an injured left internal carotid artery. Grading according to Biffl among all injuries was 17 grade II (Fig. 3), four grade III, 14 grade IV (Fig. 3), and one grade V (Fig. 4) [25].

**Fig. 5:**
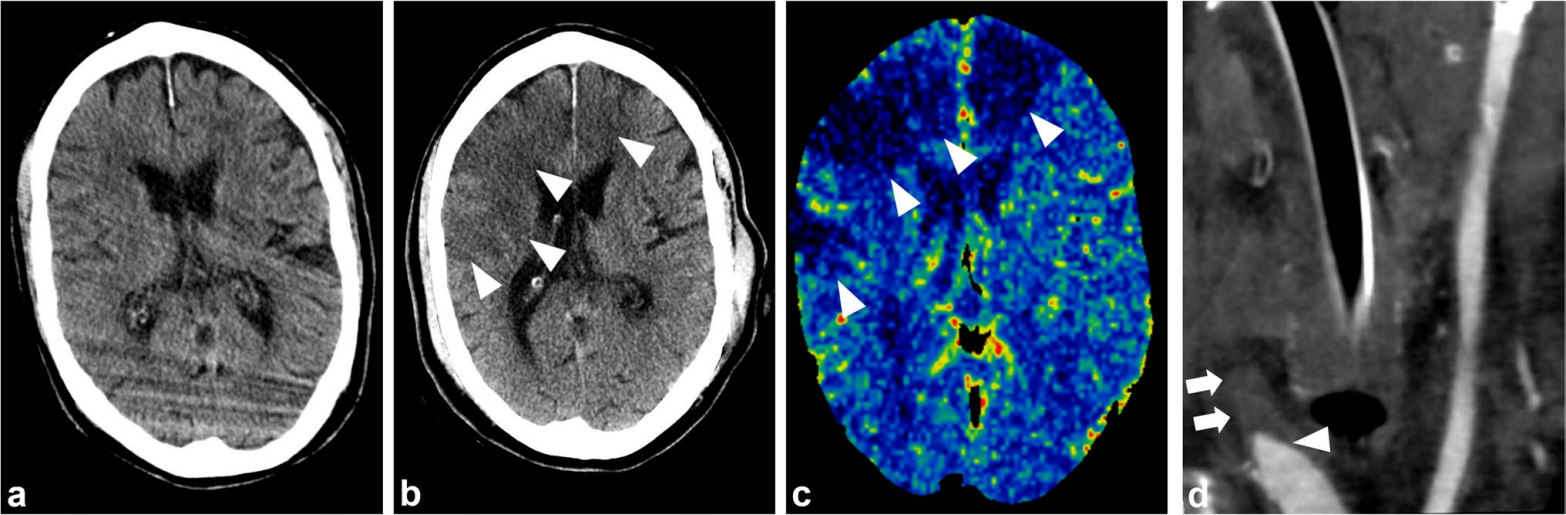
Patient aged 68 with ankylosis caused by degenerative spondylosis from the fifth to seventh cervical vertebrae. While moving with a walker, he was hit by a car going at 45 km/h, which inflicted fractures of the dens axis, ribs, and thoracic spine, in addition to multiple pelvic ring fractures. Following routine blunt trauma protocol, a non-enhanced head CT was obtained, which showed no signs of acute TBI or ischemia (**a**). Endovascular embolization of the right internal iliac artery stopped active extraperitoneal bleeding four hours after admission. Eight hours after admission, the patient fell unconscious, and an extensive acute infarction appeared in the right medial and both anterior cerebral arteries’ vascular territories (arrowheads) in a non-contrast head CT scan with 3 mm transverse slices (**b**) and on blood flow image of contrast-enhanced perfusion CT (**c**). A coronal reformatted image of 2 mm thickness from a CTA shows total occlusion of the right common carotid artery by a Biffl grade IV BCVI (arrowhead), distally to which the lumen shows no enhancement (arrows) (**d**). BCVI, blunt cerebrovascular injury; CTA, CT angiography

Six patients (21%) developed strokes visible on five head CTs and one head MRI. Four patients had acute strokes, of which three were in CTs at presentation caused by a Biffl grade IV BCVI in the right common carotid artery (Fig. 5), a Biffl grade IV BCVI in the left internal carotid artery, and a right-sided Biffl grade IV VAI. The fourth patient had acute strokes in the pons and cerebellum found in an MRI from two right-sided Biffl grade II VAIs. Two patients had strokes that emerged in the follow-up images from a right-sided Biffl grade IV VAI and a left-sided Biffl grade II VAI after two and three days, respectively.

The four patients with carotid injuries could not receive anticoagulation, and two of these developed anterior circulation strokes (Fig. 5 and 5). Contraindications for anticoagulation therapy in these four patients were TBI, massive infarction, and unconsciousness already present during admission. A severely injured patient with a TBI and a skull fracture was primarily diagnosed with a Biffl grade III BCVI in the right internal carotid artery that was initially considered non-acute. Three of the four patients with carotid injuries were severely injured and died within 30 days, a mortality rate of 75%.

Of the 26 patients with VAIs, four (15%) suffered posterior circulation strokes (Fig. 3), all of whom had cerebellar infarctions, and one patient had an additional pontine infarction. Of the 26 VAI patients, those four with strokes, and an additional six had no regular or timely anticoagulant, reaching a stroke rate of 40%.

### Trauma mechanisms and concomitant injuries

The main trauma mechanism was a ground-level fall (82%), followed by motor vehicle (9%) and bicycle accidents (5%) (Table 3). Of the 153 patients, 82 (54%) had cervical spine fractures, of which 75 were without a simultaneous skull or facial fracture. Of the 26 patients with VAIs, 24 had cervical spine fractures and one had a skull fracture, leaving only one VAI patient without a cervical, facial, or skull fracture. Of the 36 BCVIs, 26 (72%) were located on either the same or an adjacent level to the fracture. We found a total of 18 facial and 20 skull fractures (12% and 13%, respectively); of these, 12 were facial fractures without a skull or cervical spine fractures, and none had BCVIs. Of eight patients who had a skull fracture without simultaneous cervical spine or facial fracture, only one had a Biffl grade III BCVI in the right internal carotid artery.

**Table 3:** Demographics of 153 patients with ankylosis of at least three cervical vertebrae were included in the study sample from retrospectively reviewed consecutive CT angiographies of 5867 blunt trauma patients. Primary (BCVI) and secondary (acute stroke) outcomes distributed in terms of trauma mechanism, predictive (cervical spine, skull, and facial fractures), and other (TBI; findings in spinal MRI scans: spinal hematomas, damaged spinal cords, and spinal cord impingements; and etiology of ankylosis) variables. Odds ratios related to 95% confidence intervals in parentheses, and *P*-values of variables from the logistic regression model to explain the relative risk for BCVI are presented for each variable. Methods and *P*-values of statistical analysis for variables are shown to explain the association with BCVI

	A total of 153 patients with ankylosed cervical spine	29 patients with BCVIs	6 patients with acute strokes caused by BCVI	LRM between BCVI and concomitant injuries, 95% CIs in brackets	Analysis with BCVI and *P*-values
Age	IQR: 67, 5–82 Median: 75				Mann–Whitney *U* test, *P* = 0.30
Gender					*χ*^2^ test, *P* = 0.40
Male	111, 73%	22	6		
Female	42, 37%	7			
Trauma mechanism					
Ground-level fall	125, 82%	23	4		
Motor vehicle accident	14, 9%	2			
Bicycle	6, 4%	1			
Fall from over two meters	5, 3%	1			
Pedestrian struck	2, 1%	1	1		
Hit by object	1, < 1%	1	1		
Fractures					
Cervical spine fractures	82, 54%	26	4	OR 7.44 (2.22–24.98) *P* = 0.001	*χ*^2^ test, *P* < 0.001
Skull fractures	20, 13%	2	1	OR 1.05 (0.17–6.38) *P* = 0.96	Fisher’s test, *P* > 0.99
Facial fractures	18, 12%	1	1	OR 0.28 (0.03–2.47) *P* = 0.25	
Traumatic brain injury (TBI)	38, 25%	7	2	OR 1.76, (0.57–5.44) *P* = 0.33	
MRI scans	45, 29%	14	4		
Spinal cord injury	17, 11%	5	1	OR 1.37 (0.34–5.48) *P* = 0.66	
Spinal hematomas	20, 13%	6	1	OR 2.03 (0.41–10.16) *P* = 0.39	
Spinal cord impingement	25, 16%	5	1	OR 0.30 (0.07–1.37) *P* = 0.12	
Etiology of ankylosis					
Degenerative spondylosis	84, 55%	11	2		
DISH	41, 27%	11	2		
AS/Spa	18, 12%	4			
Surgical fusion	10, 6%	3	2		

*AS/SpA*, ankylosing spondylitis or seronegative spondyloarthropathy; *BCVI*, blunt cerebrovascular injury; *DISH*, diffuse idiopathic skeletal hyperostosis; *LRM*, logistic regression model; *OR*, odds ratio; *TBI*, traumatic brain injury

In 153 patients with ankylosed cervical spines, 143 head CTs revealed 38 (25%) TBIs. Forty-five patients imaged with MRI revealed either one or a combination of the following: 25 spinal hematomas (16%), 17 spinal cord injuries (11%), and 25 spinal cord impingements (16%). Of the 29 patients with BCVI, six had spinal hematomas (21%).

### Development of BCVIs

Of the 29 patients with acute BCVIs, 13 had a total of 18 BCVIs on follow-up CTAs (Fig. 1). One patient had two right-sided Biffl grade II VAIs that was followed up by MRA because of decreased renal function. In eight patients, nine (45%) VAIs improved, of which three healed completely (one Biffl grade IV and two of grade II). The sole patient with a Biffl grade V VAI received an endovascular stent without any complications. Eleven BCVIs showed no improvement, of which two grade II VAIs worsened from Biffl grade II to grade IV; the other developed an intraluminal non-occlusive thrombus. Of 15 patients who lacked follow-up imaging, three had initially missed BCVIs, two had chronic deemed BCVIs, and ten severely injured patients died within 30 days (*n* = 8) or within 2 to 3 months (*n* = 2).

### Anticoagulation treatment

Fifteen patients with BCVIs had regular anticoagulation therapy present at the moment of injury: seven with coumarin derivates, seven with platelet aggregation blockers, and one with both. Fourteen patients in this group received additional low molecular weight heparin, and one patient continued with previous warfarin treatment. Low molecular weight heparin treatment commenced in eight patients who had no regular medication before admission, and one also received clopidogrel. Seven patients had treatment without anticoagulation therapy: three had TBI, one had massive cerebral infarction and died almost immediately, two were severely injured and unstable, and one had BCVI that was initially missed. None of the patients receiving regular anticoagulation therapy had strokes.

### Original reports of BCVIs

During on-call shifts, 23 patients imaged with CTAs had 30 BCVIs of which the on-call resident recognized 25 BCVIs in 21 patients. Of the 23 patients, the daytime trauma radiologist recognized 22. Of the 21 patients diagnosed with a BCVI, three had additional Biffl grade II VAIs, which were initially missed by the resident, and two were missed by the trauma radiologist. Six patients with BCVIs had CTAs during the daytime, and two had VAIs (grade II and grade III) that were missed by the attending trauma radiologist.

### Ankylosis of the cervical spine

The most common cause of ankylosis (Table 3) was DS (84 patients, 55%), followed by DISH (41 patients, 27%), AS or seronegative spondyloarthropathy (18 patients, 12%), and surgical fusion (10 patients, 6%). Seventy-three patients (48%) had ankylosis of three consecutive vertebrae in the lower cervical spine.

Ninety-three fractured vertebrae and 25 fractured intervertebral discs were found in the 153 patients with ankylosis of the cervical spine. Forty-three fractured vertebrae or fractures running through the intervertebral discs (36%) were located above the ankylosed segment, and only six (5%) were located beneath. Sixty-nine were located within the ankylosed segments and distributed evenly to the most central vertebrae (*n* = 22, 19%), the two most cranial vertebrae (*n* = 25, 21%), and the two most caudal vertebrae (*n* = 22, 19%). In patients with ankylosis of four or fewer vertebrae, most concomitant vertebral body and intervertebral disc fractures were located superior to the level of ankylosis. In 10 patients with ankylosis of four adjacent segments to completely ankylosed cervical spine, multiple fractures were seen within the ankylosed segment.

Of the 82 patients with cervical spine fractures, 45 (55%) were diagnosed with the ankylosed cervical spine by a radiologist, and 37 (45%) had no mentions of ankylosis. Of these 37, four had mentions of unstable fractures, and eight were noticed to have ankylosis by the treating orthopedic surgeon. The majority of patients with insufficient reports of ankylosis had DS (*n* = 23, 62%), followed by DISH (*n* = 11, 30%), surgical fusion (*n* = 2, 5%), and one with AS (3%).

Cervical arthrodesis, in addition to structural changes in bone prior to the injury, was the cause of ankylosis in three patients. The surgical fixation remained intact in all patients. Two patients had posterior arthrodesis: from the third cervical vertebra to the first thoracic vertebra and from the third cervical vertebra to the second thoracic vertebra. Both patients had acute fractures of the second cervical vertebra above the fixated levels, and the latter suffered a Biffl grade IV VAI. The patient with anterior arthrodesis from the third cervical vertebra to the sixth had no fractures.

Five of seven patients had anterior disc replacements with surgical fusion, and advanced DS had two intervertebral disc implants between the fifth and sixth and between the sixth and seventh cervical vertebrae leading to consecutive ankylosis between the fifth and seventh vertebrae. One of these patients, who had an intervertebral disc fracture above the fused segments, between the fourth and fifth vertebrae, suffered bilateral VAIs, that on the right-sided being a Biffl grade IV and causing a cerebellar infarction (Fig. 3). A patient with implants between the fourth and fifth and the sixth and seventh cervical vertebrae had multiple fractures, one above and three within the ankylosed segment from the fourth to the seventh cervical vertebrae, sustaining a Biffl grade II BCVI. The seventh patient with ankylosis between the fifth and the seventh cervical vertebrae had implants between the sixth and seventh and between the seventh cervical and the first thoracic vertebra, as well as a fracture of the second cervical vertebra with a concomitant BCVI.

### Statistical analysis

Neither age (*P* = 0.30) nor gender (*P* = 0.40) was associated with BCVIs, while the association between cervical spine fracture and BCVI showed statistical significance (*P* < 0.001). In the logistic regression model (Table 3), only cervical spine fracture proved to be a significant predictive factor for BCVI, with an odds ratio of 7.44 (95% confidence intervals: 2.22–24.98, *P* < 0.001). The incidence of fractured vertebrae and intervertebral discs correlated with the number of ankylosed intervertebral discs (Spearman’s *ρ* 0.214, *P* < 0.01). Interobserver agreement on the etiology of ankylosis between Reader 1 and 2 measured by Cohen’s *κ* was substantial (0.73, *P* < 0.001), reflecting the discrepancy among 20 patients of either DS or DISH. Reader 1 and 2 agreed on all 18 patients with AS or seronegative spondyloarthropathy. Among all readers and consensus reading on BCVIs, agreements were almost perfect (0.892–0.889, *P* < 0.001).

## Discussion

Patients in our study with an ankylosed cervical spine of at least three consecutive vertebrae had an incidence of 19% for BCVI, more than seven times as high as non-specified blunt trauma patient populations, and twice as high as those with TBI [3, 5, 28]. Most injuries were located in the vertebral arteries, leading to a VAI incidence of 17%, 34 times higher than the incidence of 0.5% in general blunt trauma populations [29–31]. However, the distribution in Biffl grading among all BCVIs was similar to those in general blunt-trauma populations [5, 7, 28, 32].

Most BCVIs were on the level of the upper cervical spine with a strong association to cervical spine fractures, as previously documented [1, 4, 5, 7, 10–12, 29–31, 33]. The cranially distributed location corresponded to BCVIs of carotid arteries in general blunt trauma populations [12, 28]. Fractures of the ankylosed spine are susceptible to instability and have the strongest leverage at the craniocervical junction. The present study suggests BCVI be unlikely to occur in vertebral arteries in the absence of a cervical spine or skull fracture.

Vascular atherosclerosis, especially in the elderly, presents as uneven intimal surfaces and luminal narrowing and may cause difficulties in interpretation. Soft atherosclerotic plaques are not easy to differentiate from a Biffl grade I BCVI in trauma patients. This likely contributed to five VAIs out of 36 BCVIs being missed by attending trauma radiologists. Overly sensitive assessment resulting in unnecessary treatment should be avoided, which most likely explains the lack of grade I BCVIs in our observations. Owing to a narrower lumen, especially with atherosclerosis, vertebral arteries are more prone to progress to grade IV BCVI compared to the carotid arteries [7, 28, 29, 32].

Upon suspicion of an acute BCVI, patients had received anticoagulation treatment where possible. A BCVI in a carotid artery is considered more severe than VAI because of the larger vascular territory, and, without collateral circulation, this location results in a higher stroke rate, morbidity, and mortality [7, 25]. Although grade IV BCVI of the carotid arteries represents a small sample, the outcome tends to be high morbidity and mortality, as demonstrated by the present and previous data [2, 6, 25]. In the general trauma population, the stroke rate as a result of VAI is 0% to 24%, although Lytle et al. reported a rate of only 3% [6, 29–33]. Combining VAI patients with regular anticoagulation and those without, resulted in a stroke rate of 15%, in line with previous results [32].

Two patients with TBIs and one with a skull fracture did not receive anticoagulation treatment because their BCVIs were initially interpreted as non-acute findings, which often poses a challenge in the absence of a previous CTA for comparison. Post-traumatic patients with AS show an increased incidence of spinal hematoma, which can cause neurological deficits [34]. In the present study, 25% of patients with blunt trauma of the ankylosed cervical spine had TBI, and 20% had spinal hematomas. On follow-up imaging, 45% of BCVIs showed improvement in those with anticoagulation treatment, and one grade IV BCVI healed completely. Although spinal hematoma and TBI are contraindications for anticoagulation, patients nevertheless benefited from anticoagulation treatment.

The most common trauma mechanism was a ground-level fall (82%), which inflicted multiple BCVIs and fractures with considerably less trauma energy than those in general blunt-trauma populations [1–7, 10–12, 28–30, 33]. This mechanism is common in the elderly. A recent study reported an incidence of BCVI among patients aged 65 and over in ground-level falls and in all trauma mechanisms of 0.3% and 0.6%, respectively [35]. Compared to our sample of patients of the same age and trauma mechanisms, the incidences were 20.1% and 19.8%, respectively, indicating an increase in the relative risk up to 67- and 33-fold. Ankylosis of the spine changes the normal distribution of trauma energy and prevents motion, eventually resulting in an increased frequency and severity of fractures, as well as increased incidence of BCVIs.

Lebl et al. found a total of five patients with ankylosis caused by either AS or DISH in a cohort of 253 patients with CTA after blunt cervical spine trauma, of whom three had VAIs [36]. This suggested that these conditions are predictive for VAI. Although AS and DISH are the most recognizable causes for ankylosis and are easily identifiable on imaging studies, there are other causes, such as DS or surgical fusion of spinal segments, putting patients at risk for BCVI after blunt trauma. Failure to recognize DS as a mechanism of ankylosis likely explains the number of reports omitting to mention ankylosis in those patients. We found that DS was, in fact, the most common (55%) cause of ankylosis in our cohort.

The limitations of this study were the retrospective design and the sample size. Our hospital, as a level-1 trauma center, receives a higher amount of severely injured patients than other local centers, which probably contributed to the high incidence of concomitant injuries. Partly because of the retrospective design, only 14 out of 29 patients had follow-up imaging. The small sample size prevented reliable statistical analysis between age and ankylosis.

In conclusion, ankylosis of the cervical spine increases the incidence of BCVI up to sevenfold compared to general blunt trauma populations, affecting especially the vertebral arteries. DS was the most prevalent etiology for ankylosis, which is nevertheless less consistently included in reports than AS or DISH.

## Data Availability

Not applicable.

## Abbreviations

AS: Ankylosing spondylitis
BCVI: Blunt cerebrovascular injury
CTA: Computed tomographic angiography
DISH: Diffuse idiopathic skeletal hyperostosis
MRA: Magnetic resonance angiography
STIR: Short tau inversion recovery
TBI: Traumatic brain injury
TOF: Time of flight
TSI: Turbo spin echo
VAI: Vertebral artery injury
